# Peptide-Mediated Cellular Delivery of Oligonucleotide-Based Therapeutics *In Vitro*: Quantitative Evaluation of Overall Efficacy Employing Easy to Handle Reporter Systems

**DOI:** 10.2174/138161208786898806

**Published:** 2008-12

**Authors:** S.D Laufer, T Restle

**Affiliations:** Institut für Molekulare Medizin, Universität zu Lübeck, Ratzeburger Allee 160, 23538 Lübeck, Germany

**Keywords:** Cell-Penetrating Peptides (CPPs), nucleic acid delivery, endocytosis, fluorescence microscopy, RNAi, siRNA, steric block, splice correction.

## Abstract

Cellular uptake of therapeutic oligonucleotides and subsequent intracellular trafficking to their target sites represents the major technical hurdle for the biological effectiveness of these potential drugs. Accordingly, laboratories worldwide focus on the development of suitable delivery systems. Among the different available non-viral systems like cationic polymers, cationic liposomes and polymeric nanoparticles, cell-penetrating peptides (CPPs) represent an attractive concept to bypass the problem of poor membrane permeability of these charged macromolecules. While uptake *per se* in most cases does not represent the main obstacle of nucleic acid delivery *in vitro*, it becomes increasingly apparent that intracellular trafficking is the bottleneck. As a consequence, in order to optimize a given delivery system, a side-by-side analysis of nucleic acid cargo internalized and the corresponding biological effect is required to determine the overall efficacy. In this review, we will concentrate on peptide-mediated delivery of siRNAs and steric block oligonucleotides and discuss different methods for quantitative assessment of the amount of cargo taken up and how to correlate those numbers with biological effects by applying easy to handle reporter systems. To illustrate current limitations of non-viral nucleic acid delivery systems, we present own data as an example and discuss options of how to enhance trafficking of molecules entrapped in cellular compartments.

## INTRODUCTION

Oligonucleotide-based strategies which can be used to modulate a vast variety of cellular functions represent a promising alternative to conventional therapies (for a review see: [1,2]). Among the different oligonucleotides with therapeutic potential are aptamers, transcription factor-binding decoy oligonucleotides, ribozymes, triplex-forming oligonucleotides (TFO), immunostimulatory CpG motifs, antisense oligonucleotides, small interfering RNAs (siRNAs) and antagomirs. Nowadays, these potential macromolecular drugs are generally either relatively easily derived by rational design (e.g. antisense or siRNA) or straightforward selection processes (e.g. aptamers). One of their main advantages over protein- or peptide-based approaches comprises the high specificity for their target while being non-immunogenic. However, despite these advances, a major impediment to the development of nucleic acid-based strategies for treatment and prevention of diseases is the relatively inefficient means to effectively deliver these macromolecules into the desired target cells. Although viral vectors have been widely used to transfer genetic material into cells [3,4], they bear an inherent risk for the patient to encounter severe immunological responses or even develop cancer [5-8]. As a result of these problems much attention has been paid in recent years to the development of non-viral delivery systems. This conception includes an assortment of fairly unrelated approaches yielding various degrees of enhanced cellular uptake of nucleic acids. Currently, liposomes and cationic polymers are used as a standard tool to transfect cells *in vitro*. However, these procedures are characterized by a significant lack of efficiency accompanied by a high level of toxicity rendering them mostly inadequate for *in vivo* applications. In this context cell-penetrating peptides (see below) represent an interesting alternative as they generally are less toxic than liposomes or cationic polymers. Moreover, they are commonly better suited to transfer cargo into different cell types like non-adherent cells and primary cells, which are hard to transfect using commercially available standard protocols. The most advanced approaches in the field, which are not subject of the present article, are complex carrier systems combining vantages of assorted strategies to generate nanoparticles with better defined properties aimed towards enhanced uptake as well as intracellular trafficking in combination with cell-specific functionalities. For example, there are attempts to combine peptides with cationic liposomes [9-16] or polyethyleneimine (PEI) [17]. Other strategies are aimed towards the synthesis of high or low molecular weight branched polymers and/or peptides [18-22] or dendrimers [23,24]. Even more complex systems are particularly promising with respect to *in vivo* delivery [25-29].

In this review we will report about particular aspects of non-viral oligonucleotide delivery *in vitro*, pinpointing the current limitations, and provide quantitative means for determining where the bottlenecks of such strategies at present are. The focus of this article is recent progress in the field of peptide-mediated cellular delivery of siRNA and steric block oligonucleotides in cell tissue culture as a starting point for further developments illustrated by own experimental data. Our intention is not to provide the reader with easy solutions on how to solve the existing problems encountered with such approaches but give some hints where to start optimizing a particular approach. 

## CELL-PENETRATING PEPTIDES

The idea of using peptides as carriers goes back some twenty years when it was discovered that the HIV-1 transactivating protein Tat is taken up by mammalian cells [30,31]. A few years later, the Antennapedia homeodomain of *Drosophila melanogaster *was shown to act similarly [32]. Later on, it could be shown that peptides derived from Tat and Antennapedia as well as other proteins are capable of transporting macromolecular cargo molecules into cells [33-35]. Based on such promising results, a rapidly expanding field focusing on the so-called cell-penetrating peptides (CPPs), also referred to as protein transduction domains (PTD), began to develop. Since the first reports about Tat, a large number of naturally occurring as well as engineered CPPs have been discovered [36-42]. Table **1** gives an overview of selected “classical” CPPs. Generally, CPPs are short polycationic sequences of less than 30 amino acids that are able to translocate different cargoes (e.g. nucleic acids, peptides and even entire proteins) into cells. The only common characteristic of these peptides appears to be that they are amphipathic and net positively charged at physiological pH. Frequently the cargo is covalently attached to the CPP which can be achieved by expression as a fusion construct or by chemical coupling (for a review see: [43]). In particular cases, cargo and carrier bind each other non-covalently through mainly ionic interactions [40,44,45].

Despite the widespread interest in peptide carriers, the mechanisms underlying the cellular translocation of CPPs are poorly understood. Early work relied upon fluorescence imaging or flow cytometry analysis of chemically fixed cells to examine intracellular localization of fluorescently labeled peptides in the absence or presence of cargo. According to these experiments peptides appeared to be internalized very rapidly within minutes even at 4 °C. From such observations it was concluded that CPPs penetrate cell membranes by an energy-independent mechanism [46-50]. Although it had been reported quite early on that certain fixation procedures may cause artefacts leading to an overestimation of the cellular uptake rates [51-53] the dimension of this problem was not commonly recognized until a side by side comparison of fixed and living cells was published [54]. Based on these findings, many groups re-examined their data. However, despite considerable technical improvements, there are still puzzling controversial results concerning the exact mechanism of CPP uptake. Though in most cases endocytosis has been suggested to be the main route of internalization (Fig. (**1A**)), substantial difficulties are encountered identifying the exact pathway ([42,55] and references therein). Prior to endocytosis CPPs interact electrostatically with the extracellular matrix of the cell surface mostly through binding to negatively charged glycosaminoglycans, i.e. heparan sulfate proteoglycans [56-59]. Recent studies indicate that the uptake mechanism of CPPs can be influenced by the attachment of cargos. For example, Richard *et al.* [54,60] reported a colocalization of Tat^48-59^ with markers of clathrin-mediated endocytosis, whereas Fittipaldi *et al.* [61] found a caveolae/lipid raft-dependent process for a Tat-GFP fusion protein and Wadia *et al.* [62] described a macropinocytotic uptake pathway for a fusion construct of Tat peptide with Cre recombinase. In summary, the precise mechanism of internalization remains elusive and strongly depends on the properties of both CPP and cargo as well as on the transfection conditions and the cell lines used [63-68]. 

As opposed to the majority of CPP applications reported, which rely on covalent linkage of carrier and cargo, limiting their general use considerably as a new construct has to be generated as well as tested for any given nucleic acid cargo, we will focus in this article on a peptide termed MPGα  which forms highly stable non-covalent complexes with nucleic acids (Fig. (**1**)). The peptide is a derivative of the original MPG peptide described by Morris and coworkers [47] and differs by five amino acids in the hydrophobic part. These changes result in an alteration of the overall structure of the peptide towards a higher tendency of adopting a helical conformation [69]. Accordingly, the two peptides behave differently with respect to their interaction with artificial lipids as well as *Xenopus* oocytes [70,71] and most probably, their exact mechanism of uptake is not the same. Besides ionic interactions responsible for the initial peptide/nucleic acid complex formation, hydrophobic peptide/peptide interactions drive the maturation of large nanoparticles in a sandwich-like assembly reaction (Fig. (**1B**) and Fig. (**2**)).

## MECHANISM OF RNA INTERFERENCE

In recent years, RNA interference (RNAi) has gained a lot of interest as a tool for functional genomics studies and probably equally important as a promising therapeutic approach for the treatment of various diseases [72,73]. RNAi is a highly evolutionally conserved and specific process of post-transcriptional gene silencing (PTGS) by which double stranded RNA (dsRNA), when introduced into a cell, causes sequence-specific degradation of homologous mRNA sequences [74,75]. Mechanistically the process can be divided into two steps. An initiator step where dsRNA is cleaved by dicer, a member of the RNase III family, into 21-25 nt long small interfering RNA (siRNA) fragments [76]. In a consecutive step, these fragments are transferred to RISC (RNA-induced silencing complex) where one of the strands, the so called guide strand, serves as a molecular template to recognize homologous mRNA that is cleaved by Argonaute [77,78], a protein component of RISC. Once the guide strand is bound to RISC this complex can undergo many rounds of mRNA binding and cleavage ([79], Fig. (**3A**)). To circumvent application of long double stranded RNAs, which inevitably trigger an interferon response, it is sufficient to extracellularly supply 21 nt long dsRNAs [80,81]. Alternatively, siRNAs can be expressed endogenously using DNA vectors which code for short hairpin (sh) RNAs [82-84]. These shRNAs are than cleaved by dicer to siRNAs. Short hairpin RNA constructs have advantages over siRNA because the effects of these constructs can lead to a more stable and long-term result. On the other hand, besides the fact that shRNAs might interfere with the microRNA pathway [85,86], this strategy requires a gene therapy approach in the long run [87]. For this reason we will not cover shRNAs.

## LITERATURE OVERVIEW: CPP-MEDIATED siRNA DELIVERY

As described above, siRNAs represent a valuable tool to inhibit the expression of a target gene in a sequence-specific manner. In the following section, selected examples of CPP-mediated siRNA delivery will be presented which are summarized in Table **2**. Only a few studies describe the covalent attachment of nucleic acid cargo and peptide carrier (confer Table **2**). In one approach, simple mixing of siRNA targeted against GFP or CDK9 and Tat peptide did not generate any measurable RNAi effect whereas cross-linked siRNA-Tat^47-57^ led to a significant down-regulation of the target proteins. However, high concentrations of siRNA (about 300 nM) had to be used [88]. Both LF- and Tat^47-57^-mediated transfections resulted in a perinuclear localization of siRNA. In contrast, fluorescently labeled Tat^47-57^ without cargo was mainly found in the nucleolus, suggesting that interactions with RISC influence subcellular localization. In another approach, significant uptake of siRNAs targeted against luciferase or GFP could be observed after disulfide coupling the 5’-end of the sense strand to penetratin or transportan [89]. Compared to LF2000, slightly higher levels of transfection were achieved. Interestingly, after LF2000-mediated transfection, basal luciferase activity returned to normal levels one day earlier than after CPP-mediated transfection although the same concentration of siRNA was applied. A remarkably strong RNAi effect in hard to transfect primary neuronal cells was reported by Davidson* et al. *[90]. Here, siRNAs directed against several endogenous proteins were coupled to penetratin* via *a disulfide bond. The observed down regulation of the target proteins after peptide-mediated siRNA delivery was found to be far more effective compared to LF2000. This was in part attributed to the toxicity of the lipids. As one of the first groups to report on Tat^48-60^- or penetratin-mediated siRNA delivery *in vivo*, Moschos *et al*. showed, that intratracheal administration of the conjugates did not lead to any intensification of the knockdown of the target gene p38 mitogen-activated protein kinase in mouse lungs in comparison to unmodified non-formulated siRNA [91]. Strikingly, it was found that the peptides alone triggered a detectable decrease in target gene expression and that the penetratin-conjugate induced elevated levels of the immune markers IFN-α , TNF-α , and IL-12p40 in lung tissue. 

Besides technical difficulties arising from the syntheses of conjugates consisting of short cationic or hydrophobic peptides and highly negatively charged siRNAs, Dowdy and his group [92] present a rather critical point of view referring to previous studies with CPP-siRNA-conjugates. They claim that the successful delivery described therein is solely the result of excess free peptide, which leads to additional complexation, and thereby cellular import of the siRNA. This is in accordance with Turner *et al*. [93], who were the first to observe that careful purification of CPP-antisense-conjugates abrogates their biological effect. Among other things, this might be the reason why most of the studies reporting on successful peptide-mediated delivery of siRNAs use a non-covalent complexation approach (confer Table **2**). 

In 2003, Simeoni *et al.* [94] were the first who non-covalently complexed siRNA with the peptide MPG. At a 1:10 ratio of negative nucleic acid to positive peptide charges a decrease in luciferase activity of about 80 % was detectable in HeLa or Cos-7 cells. This effect was further enhanced to about 90 % down-regulation by a mutation in the NLS sequence of the carrier peptide (MPGΔ^NLS^), presumably due to an increased delivery to the cytoplasm, where RISC is localized. Recently, Veldhoen *et al.* [55] used a derivative of the MPG peptide for the delivery of siRNA, which will be described in the chapter “MPGα-mediated delivery of siRNA and steric block oligonucleotides”. Leng *et al.* [21] presented promising results with a prospect for cell-specific siRNA delivery. Different versions of a branched histidine/lysine-polymer (H3K8b) yielded up to 80 % knockdown of the target gene in several cell types. Structure-function studies revealed an important role of the composition of the histidine-rich domain as well as its position within the peptide and the branches for siRNA delivery, whereas size and surface charge did not have any effect. Furthermore, the toxicity was much lower than for the commercial cationic lipids Oligofectamine and LF2000. Finally, the attachment of the tripeptide RGD, an integrin-ligand, slightly enhanced siRNA delivery and turned this carrier into a cell-specific system. A similar concept has very recently been used by Kumar *et al.* [95] for a specific delivery approach into the brain. A peptide derived from rabies virus glycoprotein (RVG) interacts specifically with the nicotinic acetylcholine receptor (AchR) on neuronal cells to enable viral entry. The authors could show that the biotinylated form of the 29-amino-acid peptide (YTIWMPENPRPGTPCDIFTNSRGKRASNG) was taken up by neuronal cells. In order to transport nucleic acids with this vehicle, R_9_ was conjugated to RVG peptide. Systemic treatment of mice with siRNA in a non-covalent complex with this modified peptide promoted a highly specific cellular import of siRNA only into cells expressing AchR. Even more important, an antiviral siRNA treatment resulted in successful protection of mice against encephalitis caused by Japanese encephalitis virus (JEV). This is the first study to report on a non-toxic method to deliver siRNA across the blood brain barrier which could help to circumvent dangerous and ineffective injections into the brain. To date it presents one of the most promising tissue-specific delivery approaches which might be expandable to other *in vivo *applications. 

Along these lines, most studies today are performed with the aim of CPP-mediated siRNA delivery *in vivo*. Although many of them are already showing promising results, e.g. concerning tumor-targeting and ocular delivery [96-98], this is beyond the scope of this review and will be discussed elsewhere in this issue [99,100]. 

With the aim to increase the endosomal escape of siRNAs after peptide-mediated delivery, Lundberg *et al*. [101] rationally modified penetratin to form a CPP (termed EB1) with improved endosomolytic properties. They achieved a pH-dependent conformational change of the peptide to a higher degree of helicity by the replacement of two basic amino acids with histidines and the N-terminal addition of six amino acids. In this study, several CPPs were compared in a non-covalent approach by measuring the overall cellular uptake *via *fluorescence and biological effect of siRNA targeted to luciferase mRNA. Penetratin- as well as TP10-mediated transfection did not lead to any silencing of luciferase gene expression, despite high amounts of intracellular siRNA [101] and in contrast to previous reports using siRNA-penetratin-conjugates [90] or TP10/DNA-complexes [102]. EB1-mediated delivery of 100 nM siRNA led to approximately 50 % reduction of luciferase activity. This silencing effect was slightly better than for bPrPp and in the same range as for MPGΔ^NLS^, but still not as pronounced as for LF2000-mediated transfection of 100 nM siRNA. As it was described earlier, that addition of a pH-sensitive peptide derived from hemagglutinin (HA2) can promote endosomal escape [62], the authors linked HA2 to penetratin [101]. It turned out that although HA2-penetratin improved the silencing effect when coincubated with penetratin, EB1 was more potent than this combination of peptides. Together with confocal microscopy studies the authors concluded that the lack of biological effect after penetratin-mediated siRNA delivery is due to a lack of endosomal escape and that EB1 has a superior endosomolytic activity in comparison to HA2-penetratin. 

Endoh *et al.* [103,104] very recently presented an innovative strategy, called CLIP-RNAi (i.e. CPP-linked RBP-mediated RNA internalization and photo-induced RNAi) combining delivery of a specific RNA sequence with enhanced photoinduced release of RNA from endosomes. This goal was accomplished by fusing the U1A RNA-binding domain (RBD) to the Tat peptide and extending the siRNA with a short stretch of nucleotides specifically recognized by this RBD. These complexes were efficiently internalized but exhibited a punctuate cytoplasmic localization pattern, indicative of endosomal entrapment. However, photostimulation of a fluorophore attached to the peptide led to a redistribution of complex into the cytosol followed by efficient RNAi-mediated gene silencing.

## MECHANISMS OF ALTERNATIVE SPLICING

Human pre-mRNAs contain on average eight expressed sequences (exons) with an average length of 150 nt and up to 60 intervening sequences (introns) which can vary in length between 35 and 10,000 nt, therefore comprising up to 90 % of each transcriptional unit. In the nucleus, ribonucleoprotein complexes called spliceosomes recognize exon-intron boundaries and catalyze the precise removal of introns and subsequent joining of exons in a process called RNA splicing [105-107]. Additionally, each primary transcript can yield different mature RNAs through alternative splicing, thereby expanding the information content and versatility of the transcriptome, e.g. through the production of protein isoforms. A recent study of 10,000 human genes revealed, that at least 70 % of all multi-exon genes are alternatively spliced [108]. There are several different types of alternative splicing, amongst others affecting transcription start sites, splice sites, polyadenylation sites or even whole introns and exons. Disruptions of these intricate splicing patterns are tightly coupled with human pathophysiology, either as a determinant or a direct cause of disease or as a modifier of disease susceptibility and severity [109]. Among these diseases are β-thalassemia, cystic fibrosis, muscular dystrophies, Frasier syndrome, certain kinds of dementia and cancer. A more detailed description of the underlying mechanisms is beyond the focus of this article and can be found in a number of reviews [110-113]. López-Bigas *et al.* [114] proposed that 60 % of mutations that cause disease lead to splicing defects rather than changes in the amino acid sequence. Two common forms of mutations are depicted in Fig. (**3B**). On the one hand, a mutation in the splice donor can favor recognition of a cryptic splice donor and result in a mutant mRNA containing additional intronic sequences (part I). On the other hand, a mutation in the splice acceptor can lead to skipping of a whole exon and result in a shortened mRNA (part II). Both scenarios have been used in the context of antisense oligonucleotide-mediated approaches targeting alternative splicing. The use of antisense oligonucleotides interacting with mRNA to affect protein production goes back some 20 years [115,116]. Since then, three principle mechanisms have been exploited for this purpose (for a review see: [117,118]): (I) the oligonucleotide/RNA duplex forms a substrate for endogenous RNase H, leading to mRNA cleavage; (II) the oligonucleotide/RNA duplex prevents the productive assembly of the ribosomal complex or arrests a ribosomal complex already engaged in translation, in both cases affecting protein biosynthesis; (III) the oligonucleotide/RNA duplex alters pre-mRNA splicing in the nucleus. The following section will focus on the last approach with the aim to treat splicing disorders and give examples of possible applications for CPPs in this context. 

## LITERATURE OVERVIEW: CPP-MEDIATED STERIC BLOCK OLIGONUCLEOTIDE DELIVERY

Different forms of human β-thalassemia are caused by mutations within in the β-globin intron 2, which activate cryptic splice sites and thus lead to the formation of non-functional transcripts (Fig. (**3B** part I)). Those aberrantly used sites can be blocked by antisense steric block oligonucleotides, which leads to the synthesis of functional protein [119,120]. Kole and his group adopted this principle for the development of a splice correction assay [121]. In this model system, a firefly luciferase construct leads to the synthesis of inactive enzyme because the reporter gene pre-mRNA is interrupted by the human β-globin intron 2 containing an aberrant splice site. Upon binding of a steric block oligonucleotide, correct splicing is restored which in turn yields a functional luciferase protein. To achieve this, the oligonucleotide has to be delivered to the nucleus. Furthermore, only oligonucleotides that don’t activate RNase H are applicable [2], e.g. phosphorodiamidate morpholino oligomers (PMO, [122]), locked nucleic acids (LNA, [123]), peptide nucleic acids (PNA, [124]) or 2’-*O*-methyl-modified oligonucleotides (OMe). In addition to their inability to activate RNase H, most of these modifications confer higher affinity to the target RNA and increased resistance against enzymatic degradation than unmodified versions. Compared to RNAi-based model systems, this assay is less susceptible to side effects like cytotoxicity or off-target effects because the reporter gene activity is turned up rather than turned down. The splice correction assay has been successfully applied for the analysis of several carrier systems [24,93,125-135]. In the following section, selected examples will be presented, which are summarized in Table **3**. In contrast to many non-covalent CPP-mediated siRNA delivery approaches, efficient splice correction was only achieved with conjugates of peptide and steric block oligonucleotide. Astriab-Fisher *et al.* [128] described delivery of OMe RNA phosphorothioate oligonucleotides linked *via *a disulfide bridge to Tat peptide and penetratin. A few hours after transfection, the CPP-oligonucleotide conjugates were detected both in cytoplasmic vesicles and in the nucleus and caused a dose-dependent increase in luciferase activity. These findings are in contrast to results of Turner *et al.* [93], who could not find a biological effect for several CPP-oligonucleotide conjugates in a HeLa cell assay for Tat-mediated transactivation of the HIV-1 long terminal repeat. The authors observed vesicular uptake but no nuclear import for their highly pure conjugates. Interestingly, the rate of uptake could be enhanced by addition of free CPP to the conjugates, though still no biological activity was detected. Based on these findings, Turner *et al.* [93] concluded that these free CPPs form complexes with CPP-cargo conjugates, which play a significant role in the uptake process. This is in accordance with observations by Meade *et al.* [92] for the uptake of CPP-siRNA conjugates described above. Moulton *et al.* [136] achieved correction of missplicing at low micromolar concentrations of a R_9_F_2_-PMO conjugate but not with complexes of peptide and PMO. The steric block activity of the R_9_F_2_-PMO conjugates could be further increased with longer spacers whereas variations in the conjugation chemistry did not result in any differences. Furthermore, transfection rates were higher than for conjugates with Tat peptide, penetratin or a Tat peptide analogue. Using the HIV-1 transactivation assay mentioned above, Turner *et al.* [137] could show that most CPP-oligo-nucleotide conjugates attained biologic activity only through co-administration of the endosomolytic substance chloroquine. Fluorescence microscopy analyses revealed that this treatment released fluorescently labeled conjugates from endosomal compartments into the nucleus. Besides the addition of chloroquine, different endosome disrupting strategies have been evaluated using the splice correction assay, for example co-treatment with endosome-disruptive peptides [129] or photochemical internalization [138] (see chapter “Strategies to enhance endosomal escape”). However, the most promising results have been achieved with two newly developed derivatives of classical CPPs (reviewed in [130]). The modification of oligoarginines with non-natural, uncharged amino acids [139] led, amongst others, to the peptide (R-Ahx-R)_4_, in which Ahx represents a six-atom aminohexanoic acid spacer. Abes *et al.* demonstrated that in contrast to Tat or oligoargine, PMO-conjugates of this peptide led to dose-dependent splice correction at low micromolar concentrations in the absence of endosomolytic agents. The underlying mechanism for this superior activity is not clear yet, as the uptake of (R-Ahx-R)_4_ constructs was less efficient than the uptake of Tat or oligoarginine constructs and also involved endocytotic routes [126]. The second peptide is a derivative of penetratin, to which six arginine residues were added at the N-terminus (R_6_Pen). R_6_Pen-PNA conjugates were shown to promote efficient splice correction at low concentrations and in the absence of endosomolytic agents [127]. Again, uptake of R_6_Pen-conjugates seemed to involve endocytosis and there was hardly any difference in splice correcting activity regardless of the nature of the linker used for conjugation, e.g. a stable thioether versus a reducible disulfide linker [130].

Part II of Fig. (**3B**) illustrates a phenomenon that represents a strategy for the treatment of Duchenne muscular dystrophy (DMD). DMD is a severe progressive neuromuscular disorder caused by several different mutations in the dystrophin gene that abolish the production of functional protein [140]. Depending on the location of the mutation, the corresponding exon is skipped by covering the responsible splice sites with steric block oligonucleotides. This allows the transcription of internally deleted, but largely functional, dystrophin proteins and converts a severe DMD into a milder Becker muscular dystrophy phenotype. A more detailed description of this approach and its application in a number of animal models can be found in several excellent recent reviews [141,145]. Successful systemic delivery of splice switching oligonucleotides with or without chemical modifications (PMO, LNA, OMe) has been accomplished *via *injection of naked nucleic acids [146,147], with the help of viral vectors [148], through re-implantation of *ex vivo* manipulated stem cells [149] or in combination with CPPs [150-154]. For the latter purpose, several studies were carried out with novel derivatives of arginine-rich peptides containing different numbers of non-α  amino acids, e.g. aminohexanoic acid and/or β-alanine. These CPP-PMO conjugates showed higher serum stability, less endosomal trapping and led to efficient exon skipping in myoblasts and mice at lower dosages than the splice switching PMO alone [152,153]. Yin *et al.* [154] used a PNA-modified splice-switching oligonucleotide conjugated to Tat, a muscle specific peptide (MSP) or different functional domains of the adenovirus capsid protein VP1 (AAV6, AAV8) and examined exon skipping efficiency *in vitro* and *in vivo*. Surprisingly, both after transfection and intramuscular injection, the activity of these PNA-peptide conjugates was not significantly better than that achieved by naked neutral PNA, presumably due to endosomal trapping. 

## STRATEGIES FOR QUANTIFICATION

Intracellular trafficking represents one of the major limitations of current non-viral nucleic acid delivery approaches [155]. In other words a large percentage of intracellular cargo molecules are entrapped in vesicular compartments and thus will not trigger the desired effect. Moreover, degradation or retrograde transport might further reduce the number of active molecules. So in order to determine the overall efficacy of a given delivery approach it is essential to know the numbers of intact cargo molecules inside the cell along with the minimal numbers of molecules required to cause a particular effect. Based on such information one can easily calculate the percentage of bioactive molecules. In the following chapter we will briefly describe selected examples of variable suitability for a quantitative determination of nucleic acids in a cellular context.

In principle, either the peptide or the cargo can be labeled by a reporter group, e.g. a radioisotope [156] or a fluorophore [157]. Fluorescent peptides or cargos have been quantitatively evaluated by FACS [54] or fluorescence correlation microscopy (FCS) [158] or FRET [159]. In all cases, it is crucial to distinguish between internalized and membrane-associated signals. For this purpose, a simple wash step with just buffer is not sufficient to completely remove membrane-bound peptide/cargo-complexes [54,55]. Extracellularly bound complexes can be efficiently removed for example by enzymatic digestion with trypsin [160], acid wash [161] or heparin treatment [55,162]. Alternatively, discrimination between intra- and extracellular material is possible through chemical modification of extracellular components [163] or fluorescence quenching [164]. 

Having established that only intracellular signals are taken into account, it is still challenging to distinguish between intact and degraded forms of peptide or cargo. In two studies, a fluorescence-based quantification method was combined with either HPLC analysis [163] or “cell activity by capillary electrophoresis” [165] to verify the integrity of cargo and carrier. 

Recently, a technique to measure cellular uptake of CPPs by matrix-assisted laser desorption/ionization time-of-flight mass spectrometry (MALDI-TOF MS) was reported by the group of Burlina [166-168]. This quantification is based on the addition of an internal standard, i.e. a peptide with a stable isotope label. The method has been used to determine the amount and stability of intact internalized peptides, e.g. Penetratin, R9 and several novel CPPs, and can also be used for the quantification of peptidic cargoes, e.g. an inhibitor of protein kinase C [166]. 

Varga *et al.* [169-171] developed an “integrative systems” approach, combining quantitative experiments and computational modeling studies of vector uptake and trafficking kinetics, with the aim to take multiple potentially rate-limiting cellular and molecular processes into account. By applying their mathematical model to plasmid delivery with either Lipofectamine, several PEI-based vector formulations or an adenoviral vector, they could successfully predict experimentally observed effects and identify endosomal escape as the most important rate-limiting intracellular barrier for non-viral vectors. Recently, Zhou *et al.* [172] applied a similar strategy for the characterization of a novel lipopolymer (WLSP). This carrier shows an increased rate of endosomal escape compared to conventional PEI-based carriers.

With the aim to quantify rhodamine-labeled plasmid DNA in cellular compartments while avoiding problems arising from subcellular fractionation, like recovery and leakage, Akita *et al.* [173] developed a novel quantitative strategy, the confocal image-assisted three-dimensionally integrated quantification (CIDIQ) method. To distinguish endosomes/lysosomes and the nucleus from the cytosol, they were stained with LysoSensor DND-189 and Hoechst 33258, respectively, and sequential Z-series images were captured by CLSM. By applying this quantification method, the authors could show that due to a rapid endosomal escape, Lipofectamine Plus delivered more plasmid into the nucleus than R8 or stearylated R8. Hama *et al.* [174] used the same method in combination with TaqMan PCR to evaluate the uptake and intracellular distribution of plasmid DNA after delivery with viral as well as non-viral vectors. Due to superior cell surface binding, the efficiency of cellular uptake was significantly higher for Lipofectamine Plus than for adenovirus whereas intracellular trafficking, i.e. endosomal escape and nuclear transfer, were essentially the same. However, to achieve comparable transgene expression, 8000-fold higher intranuclear plasmid numbers were required in case of Lipofectamine Plus. This finding suggests a difference in nuclear transcription efficiency after non-viral delivery. 

In another approach, Jiang *et al.* [175] extended the 3’ end of the sense strand of a siRNA with a nuclease-resistant DNA hairpin to obtain a so-called “crook” siRNA. This modification had no effect on RNAi-mediated reporter gene inhibition and served as a primer for a filling-in reaction followed by PCR. Parameters were chosen so that the initial rate of template amplification correlates with the initial concentration of the “crook” siRNA. Under these conditions, quantification of attomolar siRNA levels per cell was possible after liposomal transfections with Oligofectamine.

A highly sensitive method was developed by Overhoff *et al.* [176] for the detection of siRNA after phosphorothioate-stimulated uptake [177] and adapted for the quantification of siRNA or steric block oligonucleotides after non-covalent peptide-mediated delivery ([55] and Laufer *et al.*, manuscript in preparation, see chapter “MPGα -mediated delivery of siRNA and steric block oligonucleotides”). This so-called liquid hybridization assay is based on the extraction of total cellular RNA and the subsequent hybridization in solution of a radioactively labeled probe which is complementary to the oligonucleotide to be detected. Finally, following PAGE analysis, absolute amounts of internalized oligonucleotide can be quantified with high accuracy down to ~10 molecules per cell using internal standards [55]. In addition to this outstanding sensitivity, no amplification step is needed and only intact oligonucleotides are taken into account. 

## MPGα -MEDIATED DELIVERY OF siRNA AND STERIC BLOCK OLIGONUCLEOTIDES – AN EXAMPLE OF A QUANTITATIVE EVALUATION

Considering the multitude of available CPPs, nucleic acid cargos and cellular as well as animal model systems, a comparison of different delivery strategies seems nearly impossible. In this context, we have for the first time undertaken a detailed side by side comparison of two different model systems using the peptide MPGα  as delivery agent. In the following paragraph we present own experimental data to exemplarily illustrate particular aspects regarding current limitations of peptide-based delivery systems. In contrast to many other CPP procedures, which rely on covalent linkage of carrier and cargo, the peptide MPGα  forms highly stable non-covalent complexes with nucleic acids, displaying binding constants in the low nanomolar range ([55], A. Trampe, unpublished data). The high flexibility of this non-covalent approach can be exploited to easily transport a wide variety of nucleic acid cargos without having to synthesize a new construct for each oligonucleotide. We have used MPGα  for the delivery of siRNAs in the context of an RNAi-based reporter system and for the delivery of steric block oligonucleotides in the context of the splice correction assay described above [121]. After MPGα -mediated transfection of a luciferase-targeted siRNA, we observed strong inhibition of reporter gene readout with an IC_50_ in the subnanomolar range [55]. After MPGα -mediated transfection of a luciferase-targeted steric block oligonucleotide (further on also referred to as ON-705), we observed a moderate up-regulation of reporter gene readout, representative of low splice correcting activity (Laufer *et al.*, manuscript in preparation). One possible explanation for the different degree of reporter gene regulation could be the different intracellular target sites, i.e. the cytoplasm for siRNAs and the nucleus for splice correction oligonucleotides. To attain more information about the subcellular localization of MPGα -oligonucleotide complexes, we performed confocal laser scanning as well as conventional fluorescence microscopy studies with fluorescently labeled siRNAs or steric block oligonucleotides. In both cases, a punctuate non-homogenous distribution of the nucleic acids inside the cells was observed. This pattern is indicative of an accumulation of nucleic acids in endocytotic vesicles, which was verified by coincubation with LysoSensor (Fig. (**4A**)). A quantitative computational analysis of fluorescence microscopy data yielded an average of approximately 50 % colocalization between endosomes and siRNA (Fig. (**4B**)). In contrast to earlier assumptions that CPPs directly traverse the lipid bilayer, it has commonly become accepted that for most peptide-cargo combinations endocytosis plays a major role in cellular uptake. As described above and in the chapter “Strategies to enhance endosomal escape” below, administration of endosome disruptive substances like chloroquine can greatly increase endosomal release of trapped nucleic acids. Chloroquine is a weak base, non-charged at neutral pH but charged at pH 5.5 [178]. It is able to pass easily through membranes in its uncharged form, but becomes protonated and accumulates within acidic vesicles in its positively charged, membrane-impermeable form. Although its exact mode of action has not yet been resolved, it is generally accepted that chloroquine works *via *prevention of endosome acidification which in turn increases the residence time of cargo within the endosomes eventually resulting in a higher probability of transfer to the cytoplasm. Fluorescence microscopy analyses in the presence of 100 µM chloroquine yielded two quite contrary outcomes. While the localization of siRNA did not change after addition of chloroquine (Fig. (**4C**)), for the steric block oligonucleotide the picture changed completely (Fig. (**4D**)). In the latter case, a diffuse fluorescence all over the cytoplasm with an accumulation of ON-705 in the nucleus could be observed. Nonetheless, considerable amounts of nucleic acid molecules were still visible as a punctual pattern, which indicates that the chloroquine treatment liberates only a certain fraction. On the whole, these qualitative observations are in full agreement with the observed biologic effects in the absence or presence of chloroquine (Fig. (**5A**)). For MPGα -mediated transfection of siRNA, even under conditions where the amount of bio-available siRNA was severely limited, only a minor increase in RNAi (ca. 30 %) was measurable. For MPGα -mediated transfection of steric block oligonucleotide, on the other hand, a dramatic increase of reporter gene up-regulation by a factor of 50 - 100 was observed. However, in both cases, the overall uptake did not change upon incubation with chloroquine (Fig. (**5B**)), which proves that the endosomolytic substance does not interfere with uptake but leads to a re-distribution of internalized nucleic acids. The underlying mechanism for the different effects triggered by chloroquine in case of peptide/siRNA and peptide/steric block oligonucleotide complexes remains unclear. Though, this is a good example that the cargo can substantially affect the properties and thereby intracellular trafficking of a particular carrier system. 

Ultimately, to assess the overall efficacy of this carrier system, we were interested to elucidate which percentage of molecules taken up after MPGα -mediated delivery is biologically active. To derive such information, the exact intracellular amount of intact oligonucleotide, the corresponding reporter signal and the minimal number of molecules necessary to trigger a specific degree of reporter gene modulation have to be known. For the quantification of internalized cargo, we adapted a highly sensitive method first described by Overhoff *et al.* [176], enabling us to detect intracellular oligonucleotide amounts down to ≥ 10 copies per cell [55]. The method is based on the liquid hybridization of a radioactively labeled probe with the corresponding oligonucleotide in cellular lysates. In this context it should be noted that a stringent heparin wash following the transfection procedure is crucial to avoid an overestimation of intracellular nucleic acid molecules due to complexes attached to the outside of the cell membrane [54,55]. In order to correlate the numbers derived from the quantification experiments with the minimal number of molecules essential to trigger the observed effect, an independent assay had to be established. The gold standard in this case is microinjection as this technique enables one to deliver definite amounts of nucleic acids with a high degree of bioavailability into the cytoplasm or the nucleus of a mammalian cell along with a low toxicity profile and great accuracy. Considering that after cytoplasmic microinjection only 12 siRNA molecules are sufficient for half-maximal inhibition of reporter gene expression, one can estimate that of the 10,000 molecules measured after MPGα -mediated transfection, only ca. 0.1 % are biologically active (Table **4**). For the splice correction assay, the numbers are different in terms of absolute numbers but the outcome remains the same. Compared to the 300,000 molecules sufficient for maximal splice correction after nuclear microinjection, of the 70,000,000 molecules required following peptide-mediated delivery only ca. 0.5 % are biologically active (Table **5**). In both cases >> 99 % of internalized oligonucleotides are most likely retained in endosomes and subsequently degraded in lysosomes after peptide-mediated delivery. Frankly, this is a sobering result and puts in numbers how much room for improvement there actually is. Though it certainly is not legitimate to generalize these findings, there are countless reports in the literature suggesting similar limitations for the majority of non-viral strategies. 

According to actual conceptions, siRNA enters a multiple-turnover pathway with one siRNA molecule capable of RISC-mediated cleavage of 50 or more mRNA molecules [79]. Even though the fate of steric block oligonucleotides is not really clear, it can be assumed that per splicing event one molecule is used up and translated into a functional mRNA molecule (e.g. single-turnover pathway). As a result the number of molecules needed to trigger an apparent effect is much higher in the splice correction assay compared to the RNAi-based reporter system (confer Table **4** and Table **5**). On the other hand, being a single-turnover pathway, the splice correction assay should be much more sensitive to even minor changes in intracellular steric block oligonucleotide concentrations whereas the catalytic nature of the multiple-turnover RNAi mechanism might mask such small variations. This is in accordance with the data described above. 

Taken together, based on the results presented as well as unpublished data (Laufer *et al.*, manuscript in preparation), the splice correction assay appears to be the superior tool for a quantitative assessment of nucleic acid delivery strategies.

## STRATEGIES TO ENHANCE ENDOSOMAL ESCAPE

As outlined above, endosomal release is one of the major rate-limiting steps for cellular delivery of macromolecules *via *cationic lipids, polyplexes and especially CPPs. In the following chapter we will present some examples of how to increase endosomal release.

Endosome-disrupting substances, like chloroquine, calcium or sucrose, were used to significantly enhance the activity of antisense PNA oligonucleotides conjugated to Tat, oligoarginines or oligolysines [125,179,180]. This effect did not result from increased uptake, but rather improved bioavailability in the cytoplasm or nucleus after endosomal escape. Takeuchi *et al.* [181] showed that by incubation of the target cells with pyrenebutyrate, delivery of arginine-rich peptides could be shifted from endocytic uptake to direct membrane translocation, yielding a rapid distribution of the peptide throughout the cytoplasm, even at 4 °C. Pyrenebutyrate acts as a counteranion and, by interacting with the positively charged peptide, increases the overall hydrophobicity, thereby facilitating a direct translocation through the lipid bilayer, as earlier shown with artificial membranes [182]. This method, which works only in the absence of a medium or serum, was successfully applied for administration of a fluorescent protein and an apoptosis-inducing peptide into dividing as well as non-dividing cells [181]. However, in general the strategies described above are not feasible for *in vivo* applications, due to high cytotoxicity or other undesirable secondary effects.

The imidazole group of histidine (His) can absorb protons in the acidic environment of the endosome, leading to osmotic swelling, membrane disruption and eventually nucleic acid escape. Accordingly, Lo *et al.* [183] modified Tat, which can bind and condense DNA through ionic interactions but has no acidic residues that can promote endosomal release, with different numbers of His residues. Highest reporter gene expression could be achieved after plasmid delivery with a Tat peptide covalently fused to 10 His residues (Tat-10H). Insertion of two additional cysteine residues into Tat-10H further enhanced stability of peptide/DNA complexes and transgene expression through formation of interpeptide disulfide bonds. Youngblood *et al.* [184] evaluated the influence of the stability of arginine-rich peptide PMO conjugates on cellular uptake and antisense activity. They could show that the stability is affected by the amino acid composition and the type of linkage to the cargo. Moreover, they found that degraded fragments could not escape anymore from endosomal or lysosomal compartments. 

Another concept makes use of photosensitive substances, which induce the release of macromolecules from vesicles by light exposure. This so-called photochemical internalization (PCI) has, in the past, been used for intracellular delivery of a large variety of macromolecules (reviewed in [185]) and, more recently, for the endosomal release of nucleic acids after delivery mediated by liposomes [186,187], polyplexes [188] or CPPs [138,189]. Shiraishi *et al.* [138] investigated the biological activity of PNAs conjugated either to Tat, R7 or KLA-peptide in combination with a PCI treatment. Depending on the peptide, nuclear as well as cytosolic antisense effects could be enhanced by up to two orders of magnitude. Similar results were presented by Folini *et al.* [189] for a PNA targeting human telomerase reverse transcriptase conjugated to Tat. In both studies, lower nucleic acid doses were sufficient, thereby reducing the probability of off-target effects. In light of encouraging data from ongoing anticancer clinical trials employing photodynamic therapy [190,191], an *in vivo* application of PCI seems feasible and will be discussed in more detail by Oliveira *et al*. [192] in this issue. Furthermore, target specificity could be increased by local illumination of cells or tissue.

Viral fusion proteins drive the fusion process between the viral membrane and the endosomal host cell membrane in a pH-dependent manner, which is required to translocate the viral genome into the cytoplasm after receptor-mediated endocytosis. Fusogenic peptides, usually hydrophobic, rich in glycine residues and found at the amino terminus of these proteins, were shown to have membrane perturbing and lipid mixing activities [193]. Many well studied representatives of this group are derived from the fusion sequence of influenza virus HA or HIV-1 gp41 [194] and have been used to improve the transfection efficiency of non-viral delivery systems [195]. 

Addition of the influenza-derived dimeric peptide diINF-7 to LF2000/siRNA complexes had no effect on the particle size of ca. 120 nm, but significantly improved gene silencing activity of siRNAs targeting the epidermal growth factor receptor or the K-ras oncogene [196]. Similar results were obtained for plasmid delivery through addition of a fusogenic peptide derived from herpes simplex virus glycoprotein H to Lipofectamine/DNA complexes [197]. To the same end, Futaki *et al.* [198] used the peptide GALA, which was specially designed to mimic the function of viral fusion sequences, together with various commercially available cationic liposomes. Although they could not detect significant differences in cellular localization, plasmid transfection efficiency was increased and liposomal dosage could be reduced.

PEI covalently modified with the HIV-1 gp41-derived peptide HGP led to a 38-fold increase of gene expression after plasmid DNA delivery and also enhanced siRNA-mediated knockdown of GAPDH by approximately 2-fold [199]. 30 % of cells incubated with PEI-HGP polyplexes showed not only the punctuate plasmid DNA staining observed with PEI polyplexes alone, but also a diffuse fluorescence throughout the cell, indicative of endosomal release of vectors. This would explain the observed increase in transfection efficiency, since the overall uptake was unaffected.

Wadia *et al.* [62] were the first to use the influenza hemagglutinin-derived fusogenic peptide HA2 in combination with a CPP. Delivery of a Tat-HA2 conjugate with increasing concentrations of a Tat-Cre conjugate enhanced reporter protein activity, presumably through an increased release from macropinosomes. The same fusogenic peptide, linked to a polyarginine-p53 conjugate, promoted release of p53 from macropinosomes and subsequent translocation to the nucleus, accompanied by an enhanced anti-cancer effect [200].

## CONCLUSIONS AND FUTURE PROSPECTS

The development of delivery systems for therapeutic oligonucleotides is a fast growing field. Owing to the enormous potential of short nucleic acids as alternative drugs such a growth is not unexpected. Besides viral vectors there is a highly diverse and constantly increasing number of non-viral systems evolving. However, despite considerable progress achieved in recent years, even the most advanced systems either lack the efficiencies required for downstream drug development or do show a substantial degree of toxicity or both. Of the many factors which limit their use, cellular uptake of the cargo/carrier complexes and subsequent intracellular trafficking to reach the target site are the most important. In addition to such essential considerations there are various additional parameters to be taken into account like serum stability, pharmacokinetic features and tissue barriers as well as target cell specificity. 

Now the question arises where to start optimizing a given delivery system. Currently, there is a clear trend towards *in vivo* testing. In principle such a development is a step in the right direction since many of the experimental data derived from artificial cell tissue culture systems with established cell lines are not applicable to the *in vivo* situation. On the other hand, it is questionable to what extent such animal experiments will eventually pay off as long as important fundamental problems remain largely unsolved. As outlined above, our quantitative studies along with microscopic analyses of siRNAs and steric block oligonucleotides using either a peptide or a commercially availably cationic lipid as carrier clearly show that less than 0.1% - 5% of molecules taken up are involved in a biological response, i.e. RNAi-mediated down regulation or splice correction-mediated up regulation of reporter gene activity. Evidently, the vast majority of internalized cargo never reaches the target. This implies that uptake *per se* is not the limiting factor here. Although there are no such detailed quantitative numbers available in the literature for other systems, there are countless reports showing that to various degrees this applies to almost any non-viral delivery approach currently available. Taken together, if one would succeed to optimize intracellular trafficking this holds the potential to boost overall efficacy by up to 3 orders of magnitude. Moreover, it is reasonable to assume that such fundamental cellular restrictions can be adequately investigated in tissue culture without the use of animal models. So it might be worthwhile to reconsider the concept of maybe premature *in vivo* testing by moving backwards one step and first optimizing the systems with regard to intracellular limitations before dealing with the next level of complexity. Accordingly, *in vitro* model systems like the ones described above for siRNAs or steric block oligonucleotides are valuable tools to study particular aspects of nucleic acid delivery. However, currently available data are based on studies using a variety of different cell lines and techniques, which renders a direct comparison of different delivery approaches impossible. In this context it would be highly desirable to introduce standardized protocols for *in vitro* testing (e.g. the splice correction system developed by Kole and coworkers [121]) together with methods for detailed quantitative analyses (e.g. the liquid hybridization protocol [55,176]). This would facilitate a direct quantitative comparison of exceedingly diverse approaches on at least the cellular level. 

One reason for the problem with intracellular trafficking of oligonucleotides arises from the mode carrier/cargo complexes are taken up by cells. Today it is well established that the majority of these complexes are taken up *via *endosomal pathways and therefore end up in vesicular compartments from which they have to escape in order to reach their target. Although there are many attempts reported to trigger endosomal escape by various strategies, they either proved to be toxic or did not achieve sustained success. Additionally, there might be a further reason for the encountered difficulties to overcome these intracellular barriers. It is not unreasonable to speculate that during evolution cells might have evolved mechanisms to avoid large amounts of foreign nucleic acids freely floating in the cytoplasm and thus safely contain them in vesicular compartments where they are eventually degraded. Alternatively they might be exported by retrograde transport. Co-evoluting viruses evidently have developed strategies to circumvent such defense mechanisms. So it might be somewhat naive to expect that a rather simple man-made carrier system is capable to efficiently overcome such an intrinsic barrier. Current developments towards more complex and elaborate carrier systems take into account such considerations. In any case it appears there is no simple solution for this problem. 

In conclusion, despite significant progress in the field of nucleic acid delivery *in vitro* as well as *in vivo*, there still is a long way to go before this will become a standard procedure in the clinic. Certain problems like endosomal escape are known for more than twenty years and still far from being resolved. In order to develop new strategies, more information about intracellular processes involved in nucleic acid trafficking is needed. Moreover, it would be desirable if the field would move from a qualitative description towards a quantitative evaluation preferentially using standardized model systems. This would allow for comparison of different approaches with one another. While animal studies are inevitable in the long run, there still is a lot of room for improvement on the cellular level. So it might be worthwhile to fathom how far we can push the different systems on this level.

## Figures and Tables

**Fig. (1):**
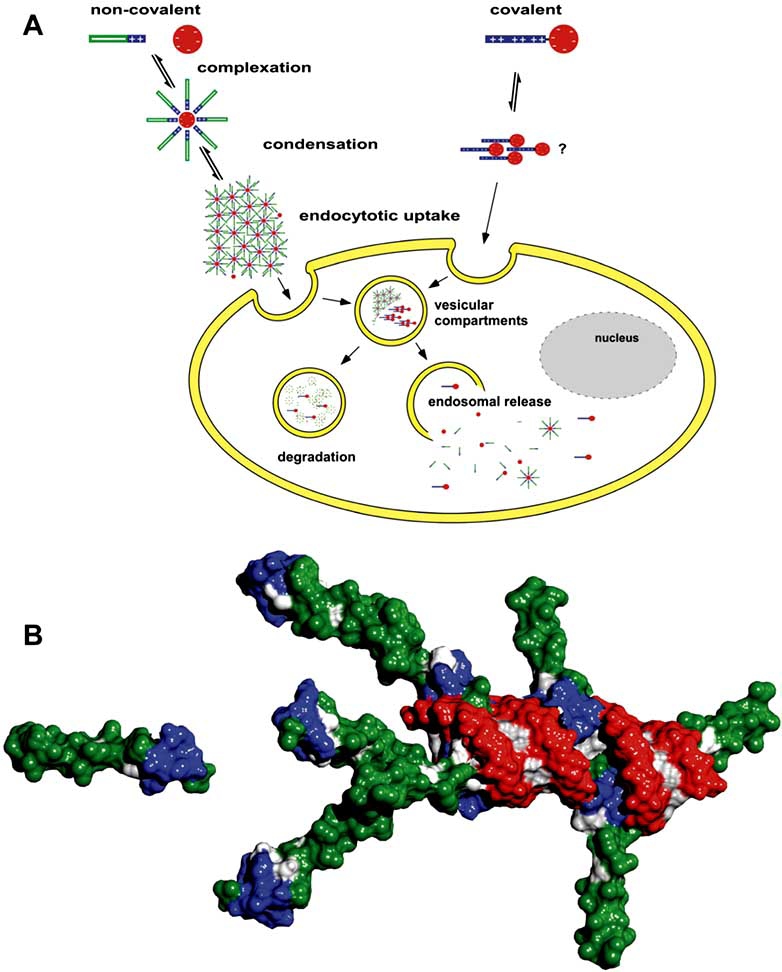
**Simplistic scheme of peptide-based nucleic acid delivery systems** (**A**). Interaction of CPP and cargo is either achieved by covalent attachment or by non-covalent complexation through mainly ionic interactions. In case of non-covalent complex formation, a further assembly of cargo/carrier complexes occurs, leading to the formation of large nanoparticles (confer Fig. (2)). In case of covalently joined molecules a similar scenario is less likely, yet cannot be excluded. Prior to the translocation process the particles attach to the cell surface by ionic interactions of positively charged CPP residues with negatively charged membrane components. Subsequently, complexes are taken up *via* an endocytotic pathway. Although less likely, direct penetration cannot be excluded and may occur simultaneously. Once inside the cell, the cargo has to escape from vesicular compartments, otherwise it eventually gets degraded in the lysosome. Red: negative charges, blue: positive charges, green: hydrophobic domains. **Three-dimensional model of MPGα/siRNA interactions **(**B**). The model was generated by iterative rigid body docking cycles of siRNA (PDB 1R9F) and peptide using the program Hex 4.2 [201]. The PDB file of MPGα was generated with the program ICM (Molsoft LLC) taking into consideration different secondary structure predictions and energy minimization protocols. Out of many docking solutions particular ones were picked for illustration purposes using the program Chimera [202]. The phosphate backbone of the siRNA is shown in red, the nucleobases in light gray. Aliphatic, aromatic and hydrophobic residues of the peptide are shown in green, positive charged residues in blue and the remaining amino acids in gray. It is assumed that formation of larger particles is driven by hydrophobic peptide/peptide interactions generating free positive charges where other siRNA molecules can interact. This eventually drives complex formation in a sandwich or mesh like assembly reaction. In principle such a scenario holds true for any given nucleic acid cargo.

**Fig. (2):**
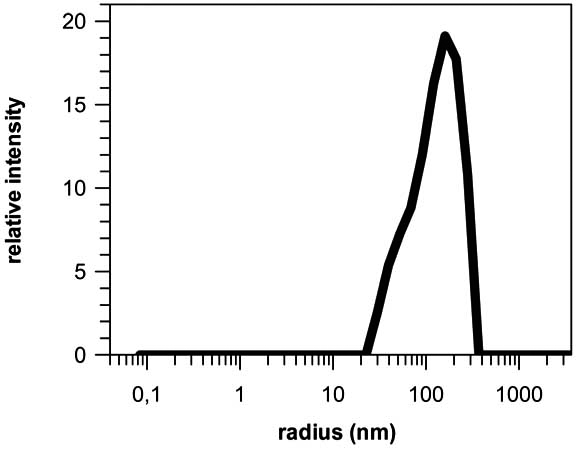
**Dynamic light scattering measurements of MPGα/siRNA complexes**. Experiments were performed with 5 µM peptide and 0.5 µM siRNA in a Tris-buffered (10 mM, pH 7.5) aqueous solution at 20°C using a DynaPro MS/X device (Protein Solutions, UK). Depending on buffer composition (e.g. ionic strength), complexes which range in size between 20 and 300 nm with an average of about 200 nm are observed.

**Fig. (3):**
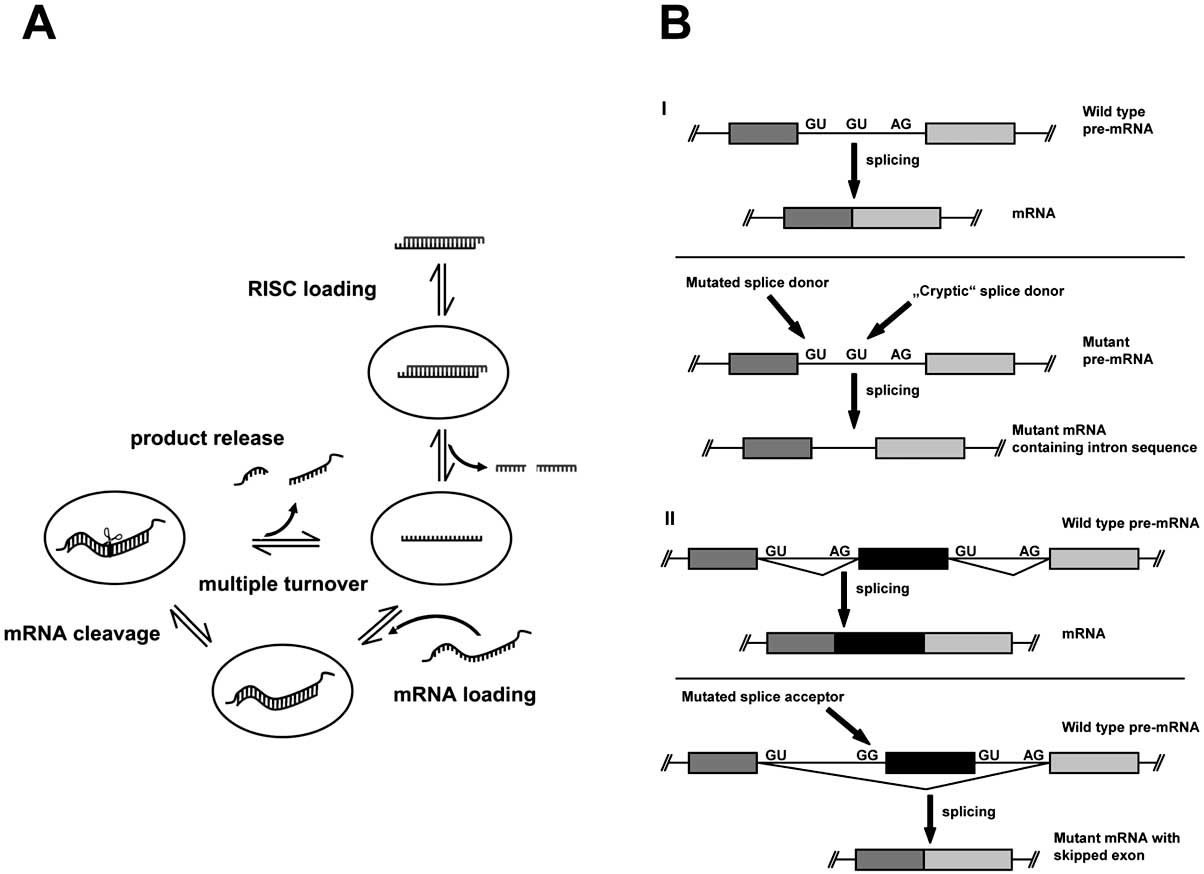
Mechanistic principles of RNAi (**A**) and splice correction (**B**). Details are given in the text.

**Fig. (4):**
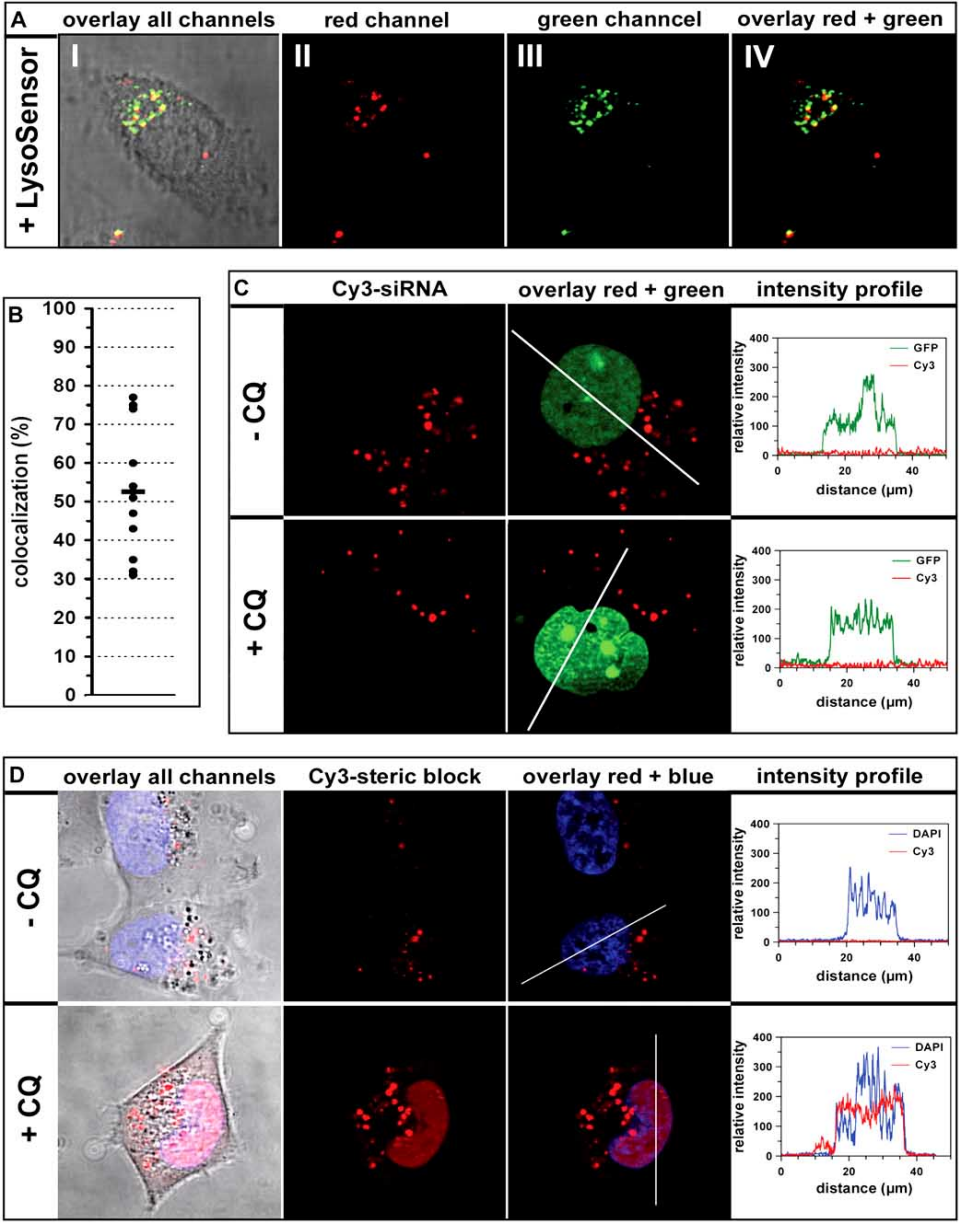
**Confocal (A) and semiconfocal (C and D) microscopic analyses of intracellular RNA localization following peptide-mediated transfection**. (**A**) Living ECV304 cells were transfected for 4 h with 4.2 µM MPGα and 10 nM Cy3-labeled siRNA. Subsequently cells were incubated with 5 µM LysoSensor (Invitrogen). (I) Overlay of the phase contrast image with all fluorescence channels, (II) Cy3-channel (siRNA - red), (III) LysoSensor channel (green) and (IV) overlay of red and green channel. Yellow dots indicate colocalization. (**B**) Quantitative analysis of siRNA/LysoSensor colocalization using the program ImageJ. For each data point 5 cells were analyzed and averaged. (**C**) Living ECV-GFP-Nuc cells [55] were transfected for 4 h with 4.2 µM MPGα and 10 nM Cy3-labeled siRNA in the absence and presence of 100 µM chloroquine (CQ).The left images show the red (siRNA) channels followed by an overlay of the red and green (nuclei) channels. The right panel shows plots of the intensity profiles for the green channel and the Cy3-channel along the line indicated in the figure to the left. (**D**) Living HeLa Luc/705 cells were transfected for 4 h with 2.5 µM MPGα and 278 nM Cy3-labeled ON-705 PTO in the absence and presence of 100 µM chloroquine (CQ). The very left images show an overlay of the phase contrast with all fluorescence channels followed by the red (steric block) and an overlay of the red and blue (nuclei) channels. The right panel shows plots of the intensity profiles for the DAPI-channel (blue) and the Cy3-channel (red) along the line indicated in the figure to the left. Cell nuclei are either stained *via* GFP-Nuc (green) or with DAPI (blue).

**Fig. (5):**
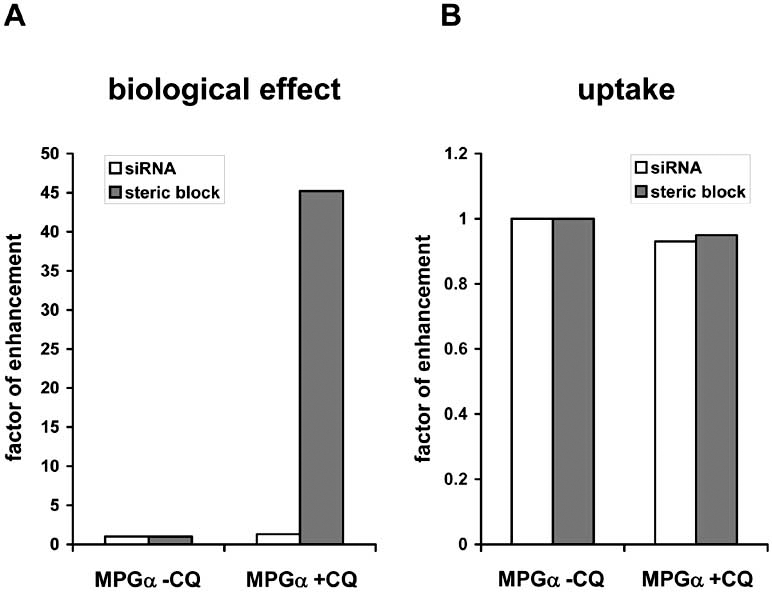
**Influence of chloroquine on MPGα -mediated delivery of siRNA and steric block oligonucleotide**. Cells were transfected with complexes of 2.1 µM MPGα and 1 nM siRNA or 2.5 µM MPGα and 278 nM steric block oligonucleotide either in the presence or absence of 100 µM chloroquine (CQ). 24 h later, RNA interference and splice correction were measured *via* reporter gene activity (**A**) and uptake was determined using the liquid hybridization protocol [55] (**B**). The factor of enhancement was calculated from three independent experiments based on the situation without CQ.

**Table 1:** Sequences of Selected “Classical” CPPs

Peptide	Sequence	References
Tat^48-60^	GRKKRRQRRRPPQ	[48]
penetratin (Antp^43-58^)	RQIKIWFQNRRMKWKK	[203]
transportan	GWTLNSAGYLLGKINLKALAALAKKIL	[204]
TP10	AGYLLGKINLKALAALAKKIL	[205]
Oligoarginine (R_8_)	RRRRRRRR	[50]
MAP	KLALKLALKALKAALKLA	[163]
MPG	GALFLGFLGAAGSTMGAWSQPKKKRKV	[47]
MPGα	GALFLAFLAAALSLMGLWSQPKKKRKV	[69]

**Table 2:** Examples for Peptide-Mediated Delivery of siRNA

CPP/Delivery System	Mode of Linkage*	Target	Cell Line	Ref.
Tat^47-57^, Tat-derived oligocarbamate)	c	EGFP, CDK9	HeLa	[88]
penetratin, transportan	c	luciferase, GFP	Cos-7, C166, EOMA, CHO-AA8	[89]
penetratin	c	Cu-ZN SOD-1, Caspase-3/-8/-9	primary rat hippocampal or sympathetic neurons	[90]
Tat^48–60^, penetratin	c	p38 MAP kinase	L929 (mouse fibroblasts), mouse lung (intratracheal)	[91]
MPG, MPGΔ^NLS^	n-c	luciferase, GAPDH	HeLa, Cos-7, HS-68	[94]
H3K8b, H3K8b(+RGD)	n-c	β-Gal, luciferase	SVR-bag4, MDA-MB-435, C6	[21]
RVG-9R	n-c	GFP, Cu-ZN SOD-1, JEV	HeLa, Neuro2a, mouse brain (intravenously)	[95]
POD	n-c	EGFP	HER 911	[96]
Chol-R9	n-c	VEGF	CT-26, mouse (intratumoral)	[97]
EB1, MPGΔ^NLS^,bPrPp	n-c	luciferase	HeLa, HepG2	[101]
TatU1A	n-c	EGFP, EGFR	CHO, A431,	[104]
MPGα	n-c	luciferase	HeLa, ECV 304	[55]
rCPP	n-c	EGFP, EF1A	HeLa, H1299	[29]
stearyl-R8	n-c	EGFP, MAP2B	primary rat hippocampal neurons	[206]
R8-MEND (siRNA/stearyl-R8 core)	n-c	luciferase	HeLa	[207]

* c = covalent / n-c = non-covalent.

**Table 3:** Examples for Peptide-Mediated Delivery of Steric Block Oligonucleotides

Oligonucleotide Cargo	CPP/Delivery System	Application	Ref.
2’-OMe phosphorothioate RNA	Tat^49-60^, penetratin	splice correction	[128]
RNA analogues	Tat^48-58^, penetratin, R_6_-penetratin, transportan, R_9_, R_9_F_2_ and further peptides	HIV-1 transactivation	[93]
PMO	R_9_F_2_, Tat peptide, penetratin	splice correction	[136]
PNA	Tat^48-58^, penetratin, transportan analogues, R_9_F_2_, R_6_-penetratin	HIV-1 transactivation	[137]
PMO	(R-Ahx-R)_4_	splice correction	[126]
PNA	R_6_-penetratin	splice correction	[127]
PMO	(R-Ahx-R)_4_AhxB	exon skipping	[152]
PMO	R_8_-derivatives containing non-α amino acids	exon skipping	[153]
PNA	Tat, MSP, AAV6, AAV8	exon skipping	[154]

**Table 4:** Summary of Quantitative Studies Concerning Peptide-Mediated siRNA Uptake

Mode of Delivery	Molecules Per Cell for Half-Maximal Inhibition
LF2000-mediated transfection	~300
MPGα-mediated transfection	~10,000
cytoplasmic microinjection	~12

Transfections were performed with 2.1 µM MPGα and 1 nM siRNA or 10 µg/ml LF2000 and 0.02 nM siRNA, i.e. in the range of the IC50 value [55]. Quantification was performed after 24 h according to the liquid hybridization protocol [55]. Molecules per cell were calculated based on the cell number seeded for transfection. For microinjection experiments, molecules per cell were calculated on the basis of the injection volume.

**Table 5:** Summary of Quantitative Studies Concerning Peptide-Mediated Steric Block Oligonucleotide Uptake

Mode of Delivery	Molecules Per Cell for Maximal Splice Correction
LF2000-mediated transfection	~7,000,000
MPGα -mediated transfection	~70,000,000
nuclear microinjection	~300,000

Transfections were performed with 2.5 µM MPGα or 7 µg/ml LF2000 and 278 nM steric block oligonucleotide, respectively, to achieve maximal splice correction. Quantification was performed after 24 h according to the liquid hybridization protocol ([55] and Laufer *et al.*, manuscript in preparation). Molecules per cell were calculated based on the cell number seeded for transfection. For microinjection experiments, molecules per cell were calculated on the basis of the injection volume.

## ABBREVIATIONS

AchR: = Acetylcholine receptor
bPrPp: = Bovine prion protein derived peptide
CLSM: = Confocal laser scanning microscopy
CPP: = Cell-penetrating peptide
CQ: = Chloroquine
DMD: = Duchenne muscular dystrophy
FACS: = Fluorescence assisted cell sorting
FCS: = Fluorescence correlation spectroscopy
GFP: = Green fluorescent protein
gp41: = Glycoprotein 41
HA: = Hemagglutinin
HIV: = Human immunodeficiency virus
IL: = Interleukin
IFN: = Interferon
JEV: = Japanese encephalitis virus
LF: = Lipofectamine™
LF2000: = Lipofectamine™ 2000
MALDI-TOF MS: = Matrix-assisted laser desorption/ionization time-of-flight mass spectrometry
MAP: = Model amphipathic peptide
MEND: = Multifunctional envelope-type nano device
NLS: = Nuclear localization sequence
OMe: 2’-*O*-Methyl
PCI: = Photochemical internalization
PEG: = Polyethylene glycol
PEI: = Polyethyleneimine
PMO: = Phosphorodiamidate morpholino oligomer
PNA: = Peptide nucleic acid
POD: = Peptide for ocular delivery
PTD: = Protein transduction domains
PTGS: = Post-transcriptional gene silencing
RBD: = RNA-binding domain
RNAi: = RNA interference
RVG: = Rabies virus glycoprotein
siRNA: = Small interfering RNA
shRNA: = Short hairpin RNA
STR-R8: = Stearyl-R8
TFO: = Triplex forming oligonucleotide
TNF: = Tumour necrosis factor
TP10: = Transportan 10
